# CPE-DB: An Open Database of Chemical Penetration Enhancers

**DOI:** 10.3390/pharmaceutics13010066

**Published:** 2021-01-07

**Authors:** Ekaterina P. Vasyuchenko, Philipp S. Orekhov, Grigoriy A. Armeev, Marine E. Bozdaganyan

**Affiliations:** 1 School of Biology, Lomonosov Moscow State University, 119234 Moscow, Russia; VasuchenkoEkaterina@mail.ru (E.P.V.); orekhov@mail.bio.msu.ru (P.S.O.); armeev@intbio.org (G.A.A.); 2 Institute of Personalized Medicine, Sechenov University, 119991 Moscow, Russia; 3 Research Center of Molecular Mechanisms of Aging and Age-Related Diseases, Moscow Institute of Physics and Technology, 141701 Dolgoprudny, Russia; 4 N.N. Semenov Federal Research Center for Chemical Physics, Russian Academy of Sciences, 119334 Moscow, Russia; 5 Department of ChemBioTech, Polytechnic University, B. Semyonovskaya 38, 107023 Moscow, Russia

**Keywords:** chemical penetration enhancers, database, stratum corneum, transdermal drug delivery, skin penetration

## Abstract

The cutaneous delivery route currently accounts for almost 10% of all administered drugs and it is becoming more common. Chemical penetration enhancers (CPEs) increase the transport of drugs across skin layers by different mechanisms that depend on the chemical nature of the penetration enhancers. In our work, we created a chemical penetration enhancer database (CPE-DB) that is, to the best of our knowledge, the first CPE database. We collected information about known enhancers and their derivatives in a single database, and classified and characterized their molecular diversity in terms of scaffold content, key chemical moieties, molecular descriptors, etc. CPE-DB can be used for virtual screening and similarity search to identify new potent and safe enhancers, building quantitative structure–activity relationship (QSAR) and quantitative structure–property relationship (QSPR) models, and other machine-learning (ML) applications for the prediction of biological activity.

## 1. Introduction

In the last few years, the number of curated open chemical databases has increased [1]. Computer-aided approaches for drug discovery are productively used for searching for novel potent compounds with biological activity. Additionally, there are databases that include formulations and additional components in pharmaceutical and cosmetics products, e.g., Formulus^®^ by CAS [2]. The cutaneous delivery route currently accounts for 8.70% of active compounds and it is becoming more common [3]. There exist several possible pathways for the skin permeation of active compounds, including intracellular penetration across the corneocytes of the stratum corneum (SC), permeation through the SC intercellular space, and incidental permeation through hair follicles, sebaceous, and/or sweat glands [4]. In this paper, we focus on the systematization of substances used for transdermal drug delivery (TDD), the method of drug delivery based on applying drug formulations onto intact and healthy skin [4]. There are different approaches that are used in TDD to penetrate the skin barrier: physical (e.g., iontophoresis, sonophoresis, electroporation, microfabricated microneedles), chemical (use of penetration enhancers), and the use of carriers (vesicles and micro/nanoparticles) [5]. Only a small part of compounds with particular physicochemical characteristics can sufficiently cut across the epidermal barriers and, in the case of medications spreading with the bloodstream, eventually reach subdermal tissue [6]. Chemical penetration enhancers (CPE) improve the transportation of other compounds across the skin layers. They achieve their effect by a number of mechanisms that depend on the chemical nature of a particular CPE [5,6,7,8,9,10]. Recently, it was demonstrated that combinations of CPEs can be designed with the desired enhancement value and cause less irritation to the skin [11]. Experimentally searching for such peculiar combinations is time-consuming, and benefits from high-throughput screening [11] and/or theoretical models.

The objective of this work is to create a chemical penetration enhancer database (CPE-DB), the first compound database of CPEs. We collected information about known enhancers and their derivatives in a single database, and classified and characterized their molecular diversity in terms of scaffold content, key chemical moieties, and molecular descriptors. The compound database is available at http://intbio.org/cpedb/.

## 2. Materials and Methods

### 2.1. CPE Database Collection

**Figure 1:**
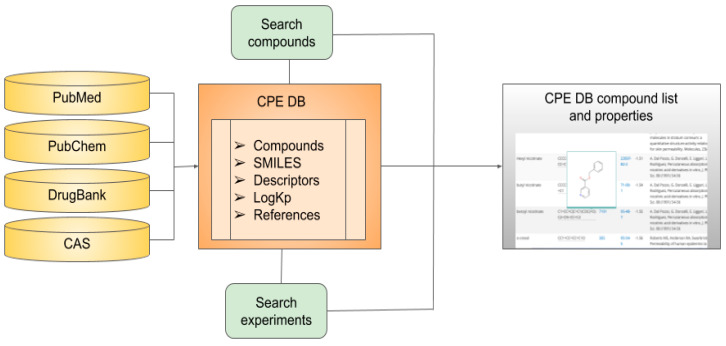
Data sources, content, and structure of chemical penetration enhancer database (CPE-DB).

The database was manually assembled on the basis of an extensive literature search (Figure 1). For searching, we used the following sources:

- PubMed (https://www.ncbi.nlm.nih.gov/pubmed/) for articles, books, and other literature sources;
- PubChem (https://pubchem.ncbi.nlm.nih.gov/) for structural information and trivial names;
- CAS (https://www.cas.org/) for CAS ID;
- DrugBank (https://go.drugbank.com) for compound status as a drug; and
- DrugInfo database (https://druginfo.nlm.nih.gov/drugportal/) for substances used as surface-active agents.

The main used publication was [12], and several papers describing different CPEs and their derivatives [13,14,15,16,17,18,19,20,21,22,23,24,25,26]. The full list of literature sources for each compound is available at http://intbio.org/cpedb/.

### 2.2. Scaffold Content and Classification

We identified the most frequent scaffolds in the compound database with scaffold-content analysis; this approach also helps to find new potent scaffolds [27]. The most frequent core molecular scaffolds were calculated as described by Bemis and Murcko [28] using the tools available in RDKit [29]. In this approach, the core scaffold was obtained by successively removing the side chains of molecules. We also used DataWarrior software [30] to classify substances by the number of functional groups in the molecules: aromatic rings, sp3 atoms, amides, amines, alkyl amines, aromatic amines, aromatic nitrogens, basic nitrogens, and acidic oxygens. In order to generate a visual representation of the chemical space of CPE-DB, principal-component analysis (PCA) was applied to the Morgan fingerprint representation of compounds in the database generated using RDKit [31,32].

### 2.3. Molecular Properties and Descriptors

All compounds included in CPE-DB were characterized by calculating the physicochemical properties: molecular weight (MW), octanol/water partition coefficient (logP), topological surface area (TPSA), number of rotatable bonds (RB), number of H-bond donor atoms (HBD) and H-bond acceptor atoms (HBA), number of aromatic rings, and the total number of ring systems. Analysis was performed using RDKit [29]. The OCHEM service was used [33] to evaluate an additional set of 499 descriptors by ChemAxon [27], which can be downloaded directly from Supplementary Materials for further analysis (Table S2).

## 3. Results and Discussion

The motivation behind the creation of a CPE database was to provide an open resource for researchers who are interested in quick access to the results of experiments and information about enhancers. As a result, a platform of known CPEs and their physicochemical properties was developed. The website contains a comprehensive profile of each compound, including its skin permeability coefficient logKp (if available), its molecular descriptors, and its current drug status. Figure 1 represents a visual overview of their content, data sources, and user-directed exploration within CPE-DB.

### 3.1. CPE-DB Structure and Content

The CPE-DB was manually established from the literature search as described in Section 2 (Figure 1). The total number of compounds currently included in the database is 649.

As the measure of human skin permeability coefficients, skin permeability coefficient logKp was provided in CPE-DB, where Kp defines the rate of penetration across the stratum corneum (usually measured in cm/h). This value is commonly used to quantitatively describe the transport of chemical compounds in the most external epidermal layer and it indicates the degree of skin absorption. The values of Kp and logKp were taken from the EDETOX database [34] and additional papers comprising lists of tested compounds [22,35,36]. For some substances, there were different Kp values available in the EDETOX database. In this case, data obtained for human samples were prioritized over other organisms. When several alternative values were present for human samples, we provided the average value in the database. When no data for human samples were available, we provided data for other organisms. In each case, the original data could be tracked down by the provided literature references.

Taken together, the fraction of compounds in CPE-DB annotated with logKp exceeded 170 CPEs (about 25% of the total number of compounds in the database). This makes CPE-DB the largest dataset of CPE permeability coefficients to date for building quantitative structure–activity relationship (QSAR) models and other machine learning (ML) applications.

### 3.2. CPEs: Mechanisms of Action and Chemical Diversity

**Figure 2:**
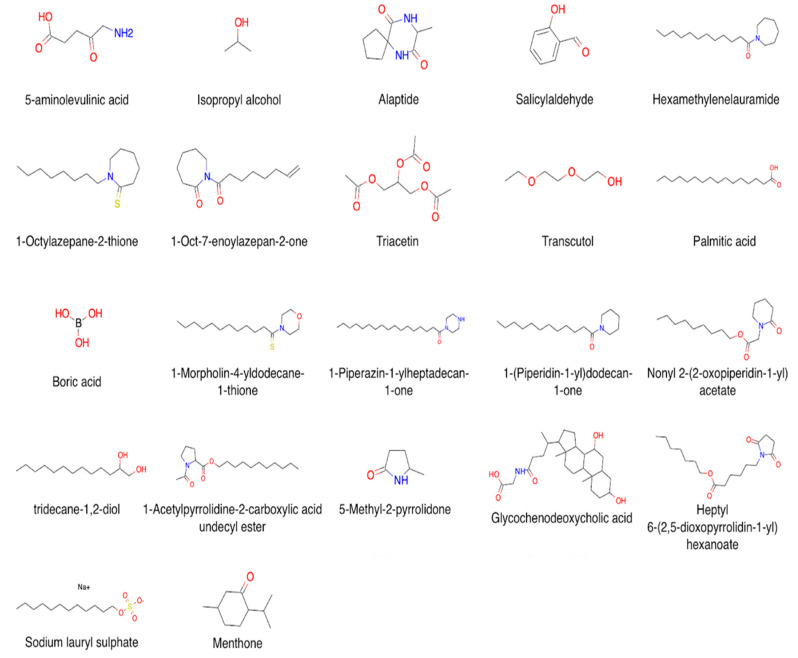
Examples of compound structures in CPE-DB.

CPEs are a chemically diverse group of compounds [4,37,38,39,40]. Their mechanisms of action are different and depend on the nature of the compound. According to [38], there are four main types of enhancers that can interact with the SC: (1) CPEs that cause swelling and increase the hydration of SC by denaturing or modifying the conformation of SC keratin (e.g., water, DMSO); (2) CPEs that affect desmosomes, specialized protein complexes responsible for cohesion between corneocytes (e.g., amino acid-based transdermal CPEs); (3) CPEs that lower the barrier resistance of lipid bilayers by affecting lipid domains (e.g., oleic acid and Azone^®^); (4) CPEs that alter the solvent nature of the SC by affecting the partitioning of active compounds or of a cosolvent into the tissue (e.g., pyrrolidones). The main criteria for CPEs [38,41] can be formulated as follows: (1) they should lack toxicity, should not cause irritations and/or allergic effects, and have no pharmacological activity; (2) their activity and the duration of action should be predictable and reproducible at the same time; (3) CPEs should promote permeability only in one direction, i.e., they should promote the transport of therapeutic agents into the body but prevent the loss of endogenous materials; (4) upon the withdrawal of CPEs from skin, its barrier properties should restore quickly and utterly; (5) they should be cosmetically appliable with a proper skin feel. The classification of CPE substances can differ depending on their mechanism of action, chemical structure, or both. We divided all enhancers in our database by chemical class combined further into groups that are typically used for the CPE classification and can be found elsewhere [12,40,42]. Thus, there are 6 groups of compounds in CPE-DB, and each group includes up to 10 classes of CPEs (Table 1, Figure 2).

Here, the division of CPEs by chemical class has some empirical aspects. Most of the compounds have several functional groups, and, on this basis, can belong to multiple classes; however, we focus on traditional and literature-based classification. In order to ameliorate this limitation, several tags were added for each compound allowing for a quick search: amides, amines, alkyl amines, aromatic amines, aromatic nitrogens, basic nitrogens, acidic oxygens. Additionally, one can search for a specific ring scaffold using its SMILES representation/trivial name.

**Figure 3:**
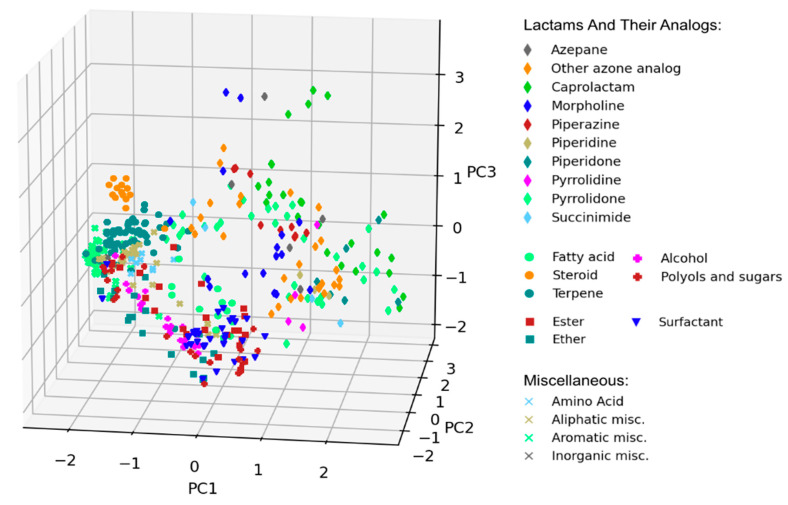
Visual representation of chemical diversity of chemical penetration enhancers (CPEs). Compounds were projected onto the first three principal components obtained using principal component analysis (PCA) and colored according to their CPE classes.

Chemical-diversity analysis of CPEs was performed with PCA on the basis of molecular topological fingerprints. All compounds from the database were projected onto the first three principal components, accounting cumulatively for ~25% of total variance in data (see Figure 3 for the 3D visualization and Figure S1 for the pairwise 2D projections).

PCA visualization confirmed that most well-defined classes of CPEs grouped together, forming distinct clusters at the PCA plot. A notable exception is a chemically diverse group of various lactams and their analogs, which occupied a broad region of the chemical space.

#### 3.2.1. Alcohols and Polyols

The alcohols group comprises compounds of lower and higher, saturated and unsaturated alcohols, while the polyols group includes noncyclic and cyclic alcohols (including sugars) with more than one hydroxyl group. Ethanol is the most used and studied enhancer and cosolvent for skin drug delivery and cosmetics. The mechanisms of action of alcohols as CPEs involve increasing the permeant concentration and affecting the lipid domains in the SC membranes [43,44,45]. Fatty alcohols also demonstrated penetration-enhancing activity [46,47,48]. In experiments with melatonin permeation [48] and saturated fatty alcohols and unsaturated fatty alcohols, a parabolic relationship between the hydrocarbon chain length (CL) of saturated fatty alcohols and the permeation enhancement of melatonin was observed for both tissue types, with the maximal permeation of melatonin observed for the fatty alcohol hydrocarbon CL of 10. Glycols can easily penetrate the skin and were assessed as a CPE in several in vitro assays [49,50,51,52]. Cyclic polyols (sugar alcohols) are also part of the group of alcohols and polyols. They are widely used as chemical enhancers [53,54,55]. Besides the modification of drug dissolution, sugars can interact with biological barriers and work as CPEs. The mechanism of action of glycols is similar to that of ethanol, but still not fully understood [45]. Propylene glycols (PGs) are often used as cosolvents. PGs increase drug permeation by improving their partition properties and reducing drug-tissue binding by the solvation of α-keratin [56,57]. Moreover, PGs affect lipids in the SC; they interact with the aqueous domains of lipid bilayers, changing the solubility of skin and increasing the drug partitioning into it [58].

#### 3.2.2. Lactams and Their Analogs

**Figure 4:**
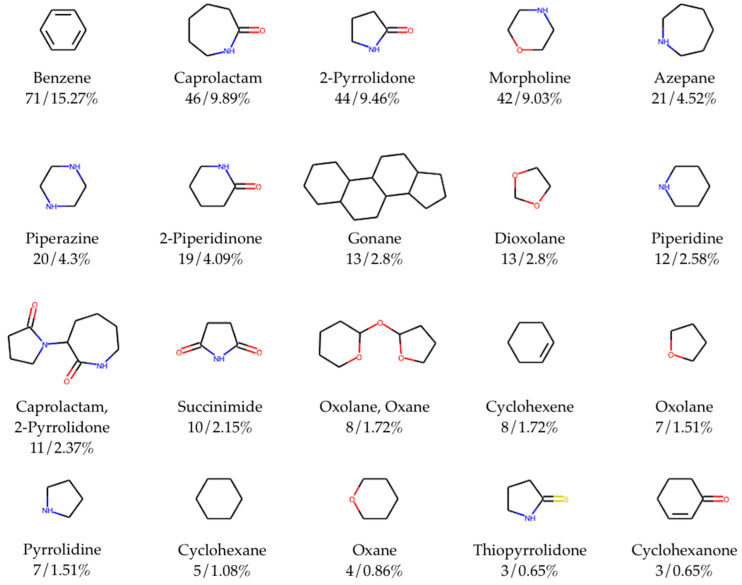
Top 20 most frequent chemical scaffolds (with trivial names of scaffolds/ring systems comprising fragments, total counts, frequency) in CPE-DB. Total number of identified unique scaffolds is 97 out of 465 compounds with cycles.

Lactams and their analogs form the largest group of CPEs in the database. Lactams are classified by scaffold, and include compounds with the azepane, caprolactam, morpholine, piperazine, piperidine, piperidone, pyrrolidine, pyrrolidone, succinimide scaffolds. The “azone other” group has modified scaffolds of Azone^®^ molecules, e.g., 1-dodecylpiperidine-2-thione or 1-dodecyl-2,7-dihydro-1H-azepin-2-one (see Figure 4 for more details). Laurocapram is the first compound that was designed as a penetration enhancer [59]. It reduces the diffusional resistance of a drug into the stratum corneum and inserts into the lipid bilayer region. Laurocapram can disrupt the highly ordered lipid patches of the bilayer [60,61]. Thus, azone molecules may exist dispersed within the barrier lipids or partition into specific membrane domains. Laurocaprams enhance the permeation of hydrophilic and hydrophobic compounds, and some peptides [62]. Pyrrolidones increase permeability by incorporation into the lipid bilayer, and amplify its fluidity by reducing resistance against the flow of substances across it [63,64]. Some pyrrolidones were already approved by the Food and Drug Administration (FDA); however, skin toxicity to N-methyl-2-pyrrolidone was reported, which implies that these compounds are not so promising for further development as CPEs [65].

#### 3.2.3. Esters and Ethers

Esters and ethers mostly include molecules with long fatty chains. Of those, 20% have a benzene scaffold, and almost half of the substances within this group were approved as drugs. Isopropyl myristate is the most common and commercially available ester used as a penetration enhancer [45]. It can penetrate the biomembrane, increasing its fluidity, which facilitates the drug flux and increases drug solubility in the SC [58,66,67]. Other examples of fatty acid esters that can be found in commercial products are glyceryl derivatives (glyceryl monolaurate and monooleate) and sorbitan monooleate [45]. Transcutol^®^ is an example of a CPE belonging to the hydrophilic ether group. The main mechanism of this CPE is to increase the partition parameter of the drug into the skin. Transcutol^®^ induces the swelling of the membrane region as it is inserted between the polar head groups of lipids, but it does not destroy the membrane structure, resulting in the increased solubility of a drug in SC [68]. The influence of Transcutol^®^ on the lipid membrane structure is still under investigation [45].

#### 3.2.4. Surfactants

Surfactants are a chemically diverse group. A typical surfactant usually consists of a nonpolar hydrophobic moiety that is a hydrocarbon chain (8–18 carbon atoms) attached to a hydrophilic part [69]. Surfactants can act differently, such as by binding to or denaturing the proteins of the skin, by solubilizing or disorganizing the intercellular lipids of the skin, by penetrating through SC, or by interacting with corneocytes [25]. In our database, surfactants are mostly represented by species with charged polar heads and with hydrophobic chains consisting of more than five carbon atoms. Neutral surfactants (e.g., amine oxides, alkanolamides, esters, and stearyl alcohol) are widely used in cosmetics, and were assigned to other classes in the CPE-DB for the reasons discussed above. Negatively charged surfactants, e.g., sodium lauryl sulfate (SLS), affect intra- and intercellular pathways in skin penetration, which can cause irritation and skin damage [45]. Additionally, SLS swells the SC, unfolds the α-keratin, interacts with and incorporates into the lipid bilayer, resulting in the formation of lamellar structures [39]. Positively charged surfactants include amines, alkylimidazolines, alkoxylated amines, and quaternary ammonium compounds. Since the SC is negatively charged, cationic surfactants can cause more defects in lipid organization than anionic and noncharged surfactants can. This makes cationic surfactants more effective, but their action causes skin irritation [45].

#### 3.2.5. Terpenes, Steroids, and Fatty Acids 

Terpenes, steroids, and fatty acids are in one group because most of them are natural compounds. Terpenes and many fatty acids are found in essential oils. Steroids, which are used as chemical enhancers, are derivatives of bile acids. This makes them normally safe for topical applications [70]. The lipophilicity of both drug and terpenes is the key factor for the enhancement effect [71,72]. The mechanism of action of terpenes is to disrupt the lipid structure of SC and to increase drug diffusivity in the case of hydrophilic drugs, and to increase drug diffusivity and drug partitioning into SC for lipophilic drugs [73,74]. The higher solubility of a lipophilic drug in the enhancer also results in its higher permeability to the SC [73].

Fatty acids are one of the most used enhancers in commercial products [45]. The higher the degree of unsaturation is, the more pronounced the enhancing effect of the fatty acid is. The cisconformation of unsaturated fatty acids leads to a higher level of disruption of SC lipids than transconformation does. A larger distance between the carboxylic group and the double bond also leads to a higher drug flux [75]. Fatty acids are often used with cosolvents as they act synergistically to enhance the penetration of a drug [16].

Steroids, which were included in the CPE-DB, are mainly bile acids [76]. Bile acids increase the fluidity of biomembranes and solubility of drugs, and promote the chemical and enzymatic stability of drug molecules [15]. 

#### 3.2.6. Miscellaneous

The group of miscellaneous compounds includes amino acids, aliphatic, aromatic, and inorganic compounds with molecular weight less than 250 Da, except for cyclodextrin and l-alanyl-l-tryptophan. Small aliphatic molecules include DMSO and similar molecules, urea and its derivatives, oxolanes, and amino acids. Small aromatic molecules include different derivatives of benzene. Inorganic enhancers are water and boric acid. The mechanisms of action on SC are different and depend on the nature of the functional group. Here, we give just a few examples. DMSO is one of the most used cosolvents in cosmetics and pharmacology, as it improves the partitioning of the active compounds into the skin [40]. DMSO enhances drug penetration by different mechanisms, including interaction with the skin lipids, keratin, and also modulating the water environment in the SC [38,45]. High concentrations of DMSO are required for effective penetration enhancement, and this leads to skin irritation [77]. Salicylaldehyde is an example of a small aromatic nontoxic enhancer [78]. Lipid-soluble and low-molecular-weight compounds such as benzene-based derivatives can pass SC with the intracellular route, which makes them perfect candidates as enhancers; however, most of them are toxic and cause irritation [19]. Water is the most natural and wildly used CPE for transdermal drug delivery. The water permeability mechanism remains unclear; the most probable explanation is suggested in [79]. This relates it to the water pools existing inside lipid bilayers leading to the lipid–water phase separation.

Cyclodextrins are cyclic sugars that are not able to penetrate the skin by themselves. However, they still are widely used in drug-delivery systems as they improve the solubility of hydrophobic drugs [4].

### 3.3. Scaffold Analysis

Scaffold analysis allows for us to classify compounds in the database and observe trends in terms of the most representable molecular scaffolds. Here, we used standard Murcko-type decomposition in order to assign chemical scaffolds to CPEs. Figure 4 shows the 20 most frequent molecular scaffolds in the CPE-DB.

Overall, the total number of identified unique scaffolds is 97 out of 465 compounds with rings (Figure S2). The top five most represented scaffolds are benzene (15.3% out of the total number of compounds containing rings), caprolactam (9.9%), 2-pyrrolidone (9.5%), morpholine (9.0%), and azepane (4.5%). The full list of scaffolds was included in the CPE-DB. SMILES and trivial names of the scaffolds were used as tags, allowing for easy access to compounds with the same scaffold.

### 3.4. Molecular Properties and Descriptors

**Figure 5:**
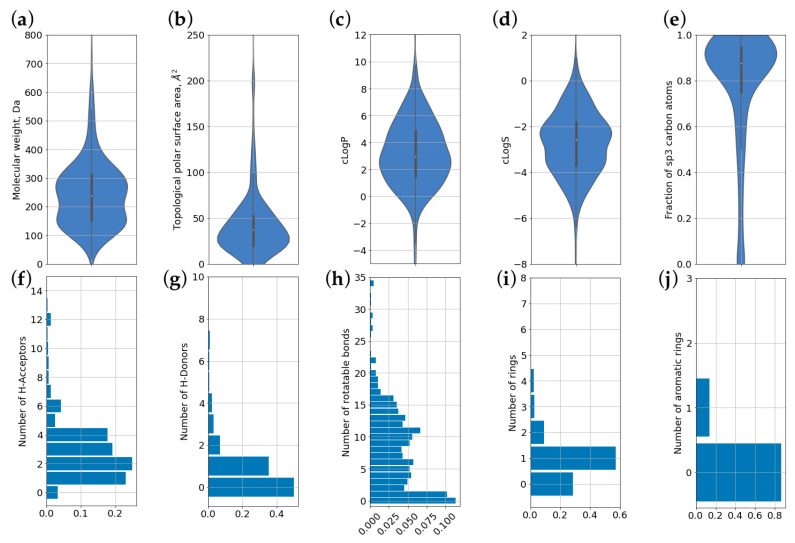
Distributions of chemical descriptors and properties for compounds present in CPE-DB. (**a**) molecular weight; (**b**) topological polar surface area (TPSA); (**c**) predicted partition coefficient (logP); (**d**) predicted solubility (logS); (**e**) fraction of carbon atoms in sp3 hybridization; (**f**) number of hydrogen bond acceptors; (**g**) number of hydrogen bond donors; (**h**) number of rotatable bonds; (**i**) total number of ring systems; (**j**) number of aromatic rings.

For all molecules, a set of descriptors was calculated that describes their physicochemical properties, as shown in Section 2. Figure 5 shows the distribution of molecules by 10 different parameters commonly used for the characterization of biologically active compounds. Data are presented as violin plots for continuous values (Figure 5a–e). This includes molecular weight, TPSA, logP, predicted solubility (logS), and the fraction of sp3-hybridized C atoms. For discrete values, histograms were built (Figure 5f–j). This includes numbers of HBA, HBD, rotatable bonds, rings, and aromatic rings. Mean, S.D., and median values of chemical descriptors and properties for CPEs are shown in Table 2.

**Figure 6:**
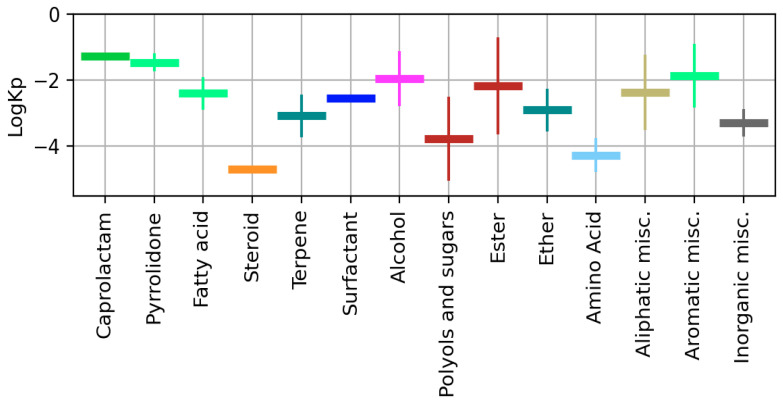
Mean value and S.D. of logKp calculated for different CPE classes.

We also provided the mean value and standard deviation of available logKp for different CPE classes (Figure 6, Table S2). Caprolactam, steroid, and surfactant groups had only one known value of LogKp, so S.D. is not shown for them. 

### 3.5. Using CPE-DB to Predict Skin Permeability of Chemical Compounds

Currently, there exist a number of computational approaches for the estimation of skin permeability of individual chemical compounds [80,81,82] that largely rely on the availability and quality of logKp data, making the CPE-DB a convenient tool for developing novel methods and for benchmarking existing ones. On the other hand, the primary aim of CPE components in topical and transdermal formulations is to modify the skin penetration of a drug achieved by a number of mechanisms, as discussed above. Therefore, the penetration of a specific drug depends on its own chemical nature, and on the chemical nature and physicochemical properties of vehicle ingredients. However, only a few attempts were made [83,84,85,86] towards a comprehensive experimental investigation of the effects of chemical mixtures on skin permeability, since such studies require extensive testing of various drug/CPE combinations. As a result, several QSAR models were proposed for the prediction of skin permeation of complex formulations [87,88], resulting in simple equations relating the logKp values of a penetrant in formulation and chemical descriptors of penetrant/vehicle [34,88,89] readily available in CPE-DB.

## 4. Conclusions

To date, CPE-DB is the first compound database of CPEs that was constructed and manually curated. The current version of CPE-DB includes 649 compounds. The compound database contains the chemical name and structure, references, chemical classification, and permeability coefficients across the skin for some of CPEs. Chemoinformatic analysis of the diversity of CPE-DB indicates that there are compounds with approved FDA status; searching for analogs might be interesting for pharmaceutical research. Similar to other chemical databases with known activities of compounds, the CPE-DB can be used for virtual screening and similarity search to identify new potent and safe enhancers, building QSAR and QSPR models, and other machine-learning (ML) applications for the prediction of chemical activity. The database is freely accessible through http://intbio.org/cpedb/.

## Figures and Tables

**Table 1:** Structure and classification of molecules in CPE-DB.

Group Name	Total Number of Compounds	Classes and Number of Compounds
Alcohols and polyols	62	Alcohol (37), polyol (25) data
Lactams and their analogs	278	Azepane (21), azone other (34), caprolactam (57), morpholine (44), piperazine (25), piperidine (12), piperidone (19), pyrrolidine (7), pyrrolidone (49), succinimide (10)
Esters and ethers	53	Ester (31), ether (22)
Surfactants	39	Surfactant (39)
Fatty acids, terpenes, steroids	112	Fatty acid (31), terpene (67), steroid (14)
Miscellaneous	105	Amino acid (8), aliphatic misc. (41), aromatic misc. (54), inorganic misc. (2)

**Table 2:** Average values of chemical descriptors and properties for compounds present in CPE-DB.

Property	Mean	S.D.	Median
Molecular weight, Da	251.24	139.55	239.4
TPSA, Å^2^	43.43	42.07	37.3
logP	3.15	2.69	2.95
logS	−2.75	1.48	−2.59
Fraction of carbon atoms in the sp3 hybridization	78%	27%	88%
Number of H-bond acceptor atoms (HBA)	3.02	2.78	2.0
Number of number of H-bond donor atoms (HBD)	0.88	1.55	1.0

## Data Availability

The database is freely accessible through http://intbio.org/cpedb/.

## Supplementary Materials

The following are available online at https://www.mdpi.com/1999-4923/13/1/66/s1, Figure S1: Visual representation of the chemical diversity of CPE. Pairwise 2D projections of CPE compounds onto the first three principal components obtained using PCA and colored according to their CPE classes, Figure S2: Frequency of the Murcko scaffolds found among CPE compounds in the CPE-DB. Table S1: Mean value and S.D. of logKp calculated for different CPE classes, Table S2: Set of 499 descriptors for compounds of CPE-DB.
