# Supplementary material for: CPE-DB: An Open Database of Chemical Penetration Enhancers
Source: Pharmaceutics. 2021 Jan 7;13(1):66. doi: 10.3390/pharmaceutics13010066 (PMC7825720; doi:10.3390/pharmaceutics13010066)
Supplement: Supplementary file 1 [file pharmaceutics-13-00066-s001.zip › Figure S1, Figure S2, Table S1.docx]

Supplementary Materials: CPE-DB: An Open Database of Chemical Penetration Enhancers


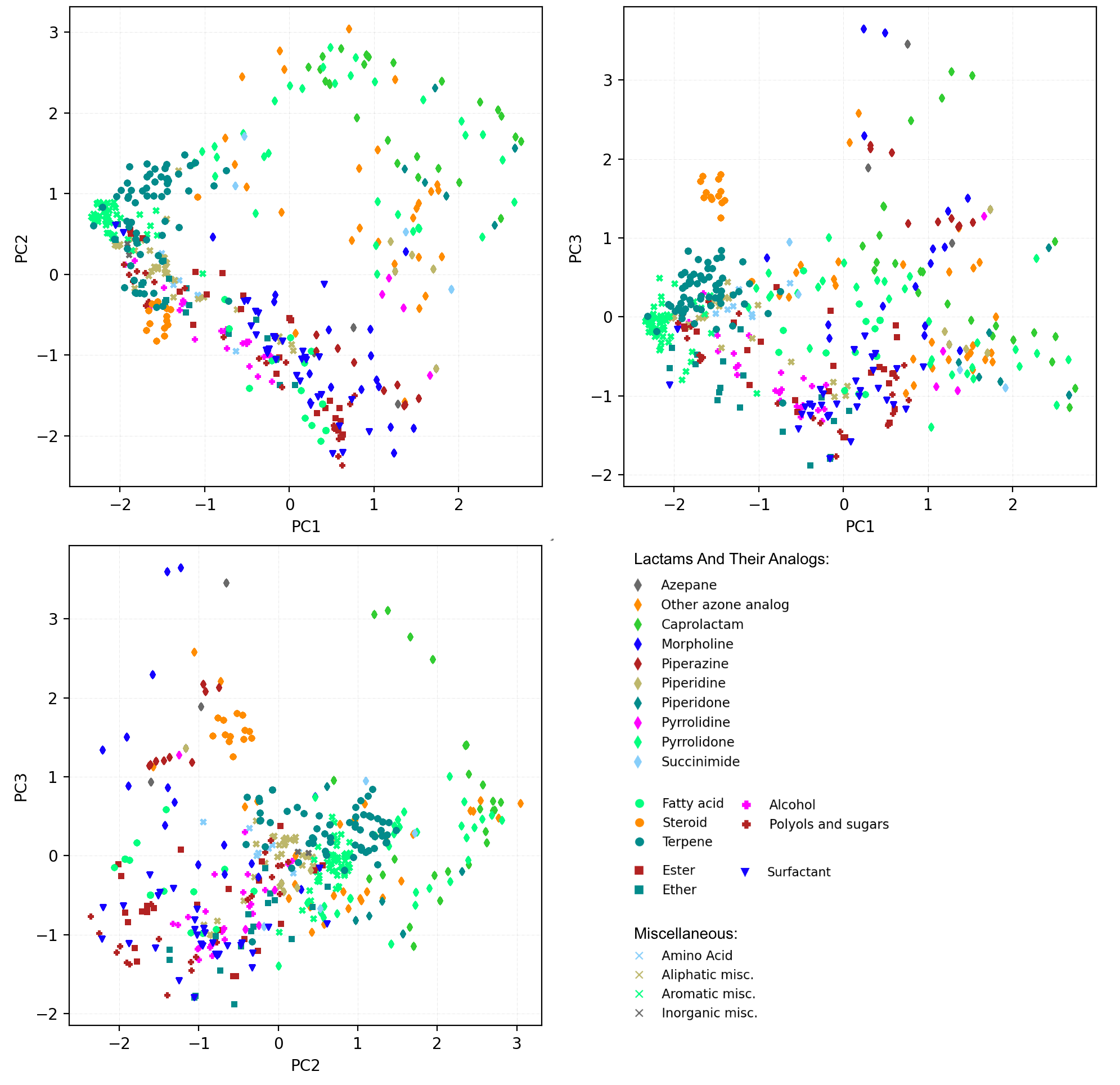


**Figure S1. Visual representation of the chemical diversity of CPE.** Pairwise 2D projections of CPE compounds onto the first 3 principal components obtained using PCA and colored according to their CPE classes.

**Table S1.** Mean value and S.D. of logKp calculated for different CPE classes.

| **Class** | **Mean logKp** | **S.D.** |
| --- | --- | --- |
| Amino Acids | -4.27 | 0.52 |
| Alcohols | -1.95 | 0.83 |
| Aliphatic Misc. | -2.37 | 1.14 |
| Aromatic Misc. | -1.86 | 0.97 |
| Caprolactams | -1.28 | - |
| Esters | -2.17 | 1.47 |
| Ethers | -2.9 | 0.64 |
| Fatty Acids | -2.4 | 0.5 |
| Inorganic Misc. | -3.28 | 0.42 |
| Polyols and sugars | -3.76 | 1.27 |
| Pyrrolidones | -1.46 | 0.27 |
| Steroids | -4.69 | - |
| Surfactants | -2.54 | - |
| Terpenes | -3.08 | 0.66 |


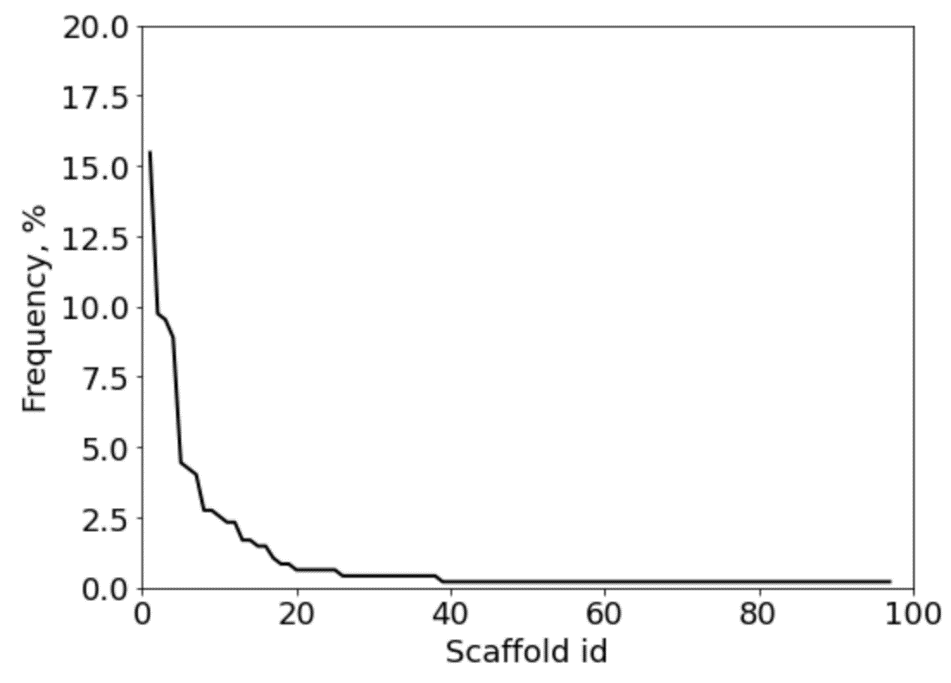


**Figure S2.** Frequency of the Murcko scaffolds found among CPE compounds in the CPE-DB.
